# Family-Run Pig Farms: Research and Extension Activities for Parasite Control in a Municipality in the State of Rio de Janeiro, Brazil

**DOI:** 10.3390/pathogens11090971

**Published:** 2022-08-26

**Authors:** Camila Class, Renato Silveira, Priscila Fialho, Letícia Silva, Lucas Lobão, Maria Regina Amendoeira, Alynne Barbosa

**Affiliations:** 1 Department of Microbiology and Parasitology, Biomedical Institute, Fluminense Federal University, Niterói 24210-130, RJ, Brazil; 2 Department of Morphology, Biomedical Institute, Fluminense Federal University, Niterói 24210-130, RJ, Brazil; 3 Laboratory of Toxoplasmosis and Other Protozooses, Instituto Oswaldo Cruz, Fundação Oswaldo Cruz, Rio de Janeiro 21040-900, RJ, Brazil

**Keywords:** pigs, gastrointestinal parasites, risk factors, rural extension

## Abstract

The parasites infesting pigs and pig farmers on family farms in Rio de Janeiro city, Brazil, were analyzed, and extension activities were carried out to impart information about parasites. Between 2020 and 2021, fecal samples were collected from 180 pigs as well as ear scrapings from 142 pigs. In addition, 34 stool specimens from farmers and their families were analyzed. The collected material was processed by parasitological techniques. Parasites were detected in 86.1% of the pigs, forms of phylum Ciliophora (70.5%), strongylids (56.7%), *Strongyloides ransomi* (44.4%), coccidia (38.3%) and *Ascaris suum* (32.2%). *Sarcoptes scabiei* var. *suis* were identified in 3.5% of the pigs. An analysis of infections by age group revealed that the general frequency of phylum Ciliophora and strongylid parasites was statistically significant. Other factors also associated with the frequency of the parasites included the type of food fed to the pigs, cleaning of the facilities, care of piglets and type of facility. The frequency of intestinal parasites of farmers themselves was low; however, the high rate of parasite infections detected in pigs highlighted the precarious conditions of hygiene of the farms. Lastly, the farmers’ participation in extension activities was a step forward in actions to improve their farm management.

## 1. Introduction

Population growth associated to the COVID-19 pandemic health crisis has caused the impoverishment of populations, particularly those living in developing countries such as Brazil [1]. In these countries, food has become increasingly expensive, especially the rising price of animal products such as beef, which is one of the main sources of protein [2]. With the increase in worldwide demand for meat, fast-growing species with efficient feed conversion rates such as pigs are likely to account for a major share in the growth of the livestock subsector. Pig farming is an important activity that provides opportunities for generating income for small-scale farmers [3].

In addition, pigs offer the advantages of high fertility rates, early maturity, short generation interval, small space required to raise them, and their ability to produce maximally under varied management conditions [4]. The growth in pig production contributes substantially to national gross domestic product (GDP) and general economic growth, providing an additional source of animal protein for human consumption, generating employment and reducing poverty [5].

Investment and research in pig farming have positioned Brazil in fourth place in the ranking of world pork production. In 2021, more than four thousand tons of pork meat were slaughtered [6]. The regions with the largest concentrations of industrial farms are the South, Center-West and Southeast of the Brazil, the latter being where Rio de Janeiro state is located. According to estimated data from IBGE (2017), Rio de Janeiro state has 7171 heads of pigs and properties located in several cities [7]. Furthermore, in Brazil, a large part of pig production is concentrated on small farms, which raise these animals as a source of income and subsistence. Generally, this type of pig farming involves simple systems with little financial and technological investment. Approximately 46.5% of the 5.8 million farms in Brazil engage in pig raising, typically employing family labor [8]. Although there are official data on industrial properties, the same does not occur with smaller family-type creations. Family-type swine farming in Brazil is difficult to quantify and analyze due to the lack of data and information, as if these properties did not exist [9].

In family pig farming, health problems can be attributed to a variety of biological agents, including parasites. The main negative effects of parasitic diseases in pigs are economic losses for producers, such as reduced feed conversion, reduced fertility, low number of piglets born and weaned, low weight at birth and at weaning, as well as losses resulting from high discard rates in slaughterhouses [10,11,12,13]. Moreover, pigs are considered the main reservoirs of various gastrointestinal parasites with zoonotic potential, including *Balantioides coli*, *Entamoeba suis*, *Blastocystis* sp. and *Ascaris* spp. [14,15]. This health problem could be mitigated and even overcome in the long term if scientific technical support were provided to these small producers through continuous work in the area of rural extension [16,17].

However, there is still a paucity of studies in the scientific literature about research of parasites on family properties, where extension activities are performed to support these small producers in pig farming management. It should be noted that on this topic, only one scientific article in the literature we consulted associates research with rural extension, and the authors belong to our research group [17]. To improve the activities carried out by extending the study initiated to other locations, this study aimed to analyze the frequency of parasites detected in pigs, relating their positivity rates with risk factors, and to perform the same analysis on human fecal samples from pig farmers on family farms to detect mainly parasites with zoonotic potential, associating the information garnered in this research with extension activities in order to disseminate information about parasite control.

## 2. Results

The number of pigs on each of the ten family-owned pig farms participating in this study varied from 3 to 41, making a total of 180 female and male pigs of different age groups. A combination of the results of the qualitative laboratory techniques for the examination of feces revealed an overall parasite positivity rate of 86.1%. Protozoa were detected slightly more frequently than helminths, the former being identified in 140 (77.8%) animal fecal samples and the latter in 126 (70%). The positivity rate for gastrointestinal parasites at all the family farms included in this study exceeded 85% (Table 1).

**Figure 1:**
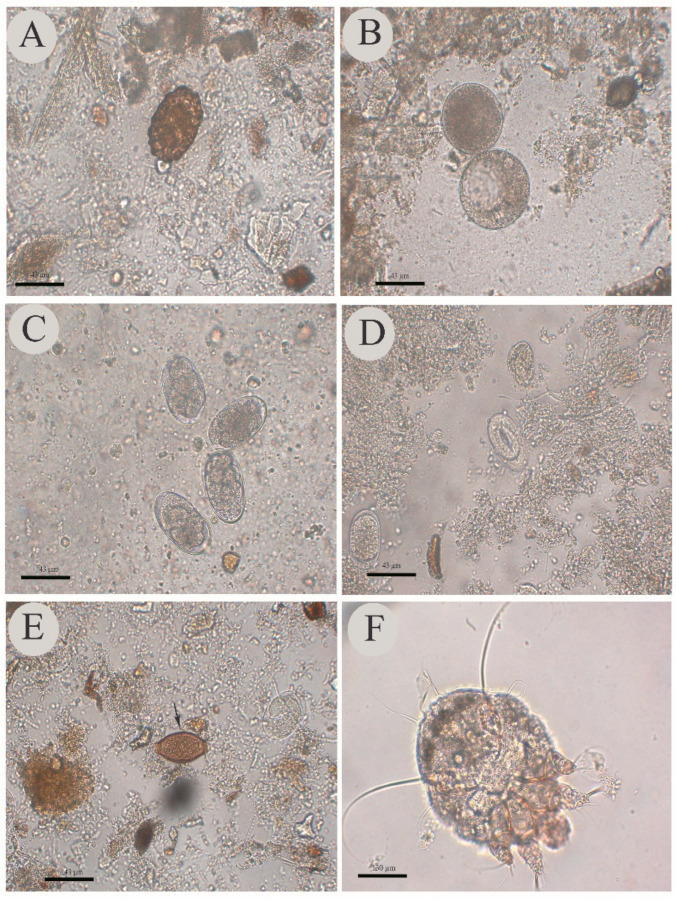
Some of the parasites detected in pigs. (**A**) *Ascaris suum* egg. (**B**) Cyst of the Phylum Ciliophora. (**C**) Strongylid eggs. (**D**) *Strongyloides ransomi* eggs. (**E**) *Trichuris suis* egg. (**F**) Adult form of *Sarcoptes scabiei* variety *suis.* (**A**–**E**) photos at 400 × magnification (43 µm bar). (**F**) 100× magnification photo (130 µm bar).

The parasites most frequently detected in the fecal samples of these animals were cystic and trophozoites forms of the phylum Ciliophora, strongyle eggs, *Strongyloides ransomi* eggs and larvae, oocysts of non-sporulated coccidia, and eggs of *Ascaris suum* and *Trichuris suis*, whose positivity rates were statistically significant (*p* ≤ 0.05) (Table 1). Some parasites detected are shown in Figure 1. Parasites that were also detected, albeit without significant relevance, were amoeba and *Blastocystis* sp. cysts, nematode larvae and *Capillaria* sp. eggs, (*p* > 0.05) (Table 1).

Forms compatible with arthropods were detected in 13 (9.1%) of the 142 biological samples of ear scrapings, including arthropod egg, forms of *Sarcoptes scabiei* variety *suis* and *Demodex phylloides* compatible with adult, larva and/or nymph forms but whose diagnostic frequency was not statistically significant (*p* > 0.05) (Table 1). Adult forms of *Sarcoptes scabiei* variety *suis* are shown in Figure 1.

The farm owners’ answers to the questionnaire revealed that 90% of them raised crossbred pigs, their premises had no water sources for the pigs to cool off, and their pig sheds were roofed with fiber cement tiles, galvanized roofing sheets or PVC tiles. However, they all reported routinely spraying the animals down with a hose to help them keep cool. More than 80% of the farmers reported not treating their pigs with anti-ectoparasitic drugs and using cleaning tools only to clean the pigsties. All the owners stated that their pigs had ad libitum access to drinking water, they had other animal species in their farms, treated their pigs with anthelmintics and never found blood in their feces. Moreover, none of them had ever carried out a sanitation break protocol on the farm or used a flamethrower, detergents or disinfectants to sanitize the sheds. Eighty percent of the farmers stated their farms had pen-type enclosures; they fed their pigs twice a day, stored pig feed on the farm, and mating was allowed to occur naturally. Moreover, 80% reported that they had already observed rodents in their farms and that they did not change their clothes after handling the animals (Table S1).

From the 180 pigs included in the fecal parasite survey, 109 were females and 71 were males. Females showed a parasite positivity rate of 87.1% and males showed a parasite positivity rate of 84.5%. As for the statistical relevance of the parasite taxa, the univariate analysis revealed that only *Strongyloides ransomi* showed a significant difference between the sexes (*p* ≤ 0.05), and that eggs of these parasites were detected mainly in the feces of females. With regard to age groups, the highest frequency of gastrointestinal parasites was detected among pigs in the growing phase, which was followed by the finishing and nursery phases. The univariate analysis indicated a statistically significant difference between the pig age groups and the overall gastrointestinal parasite positivity rate as well as between the diagnosed forms compatible with phylum Ciliophora and strongylids (*p* ≤ 0.05), which were mainly detected in the growing and termination phases, respectively (Table 2). The univariate analysis of screening for taxa of specific parasites indicated that other variables were also statistically significant in the overall gastrointestinal parasite positivity rate, involving both general information about each farm and its pigs, as shown in Table 2, and pig hygiene and health management (Table 3).

Information identified as statistically significant in the univariate statistical tests was later analyzed jointly in the final logistic regression model for each parasite taxon and for the overall gastrointestinal parasite positivity rate. Based on this analysis, it was found that only the age of the pigs remained statistically significant in the overall parasite positivity rate. Pigs in the growing age group were four times more likely to be parasitized than those in the other age groups. This age group also showed a higher odds ratio for infection by protozoa of the phylum Ciliophora and by strongyle helminths. The type of food fed to the animals, i.e., leftover human food and agricultural waste, wheat and/or barley bran; the behavior of not washing the facilities and of using straw bedding in the pens, as well as the indifferent or insufficient care given to piglets at birth, were statistically associated with the animals’ infection by coccidia and *S. ransomi*. Several variables in the univariate analysis were relevant in the frequency of *Ascaris suum* and *Trichuris suis* in family properties. However, only the type of facility for *Ascaris suum* was a variable associated with infection, highlighting the risk of collective bays with cement and wood walls.

In other words, pigs raised in pens with cement and wood walls were almost nine times more likely to be parasitized than those housed in other types of facilities. Lastly, with regard to strongylids, not only the pigs’ age but also the fact that pig sheds were not washed and had cement or concrete floors was directly associated with infection in this study (Table 4).

Among the 34 people that handed over their stool samples for analysis, 24 were male and 10 were female. Only one female participant was a pig producer (owner) and caretaker. The average age of the participants was 37 years, the youngest being a 1-year-old baby in arms, the son of a producer, and the oldest an 88-year-old pig farmer/caretaker. Parasite forms were detected in stool samples from six participants, five of which consisted of *Entamoeba coli* cysts and one was an adult form of *Enterobius vermicularis*.

According to the information garnered from the questionnaires answered by the pig farmers and their family members, more than 75% of them reported that they had performed a stool exam in the past, used antiparasitic drugs, had never noticed blood in their stool, did not treat the water used in their homes, and consumed well-cooked pork. More than 50% of the farmers and their families had orchards, and they cultivated vegetables and legumes on their farms for their own consumption. Most of the participants reported raising pigs for their own consumption. However, about 38% also reported raising pigs as a source of income, carrying out small exchanges and sales (Table S2).

In general, at least one person on each farm participated in all the proposed extension activities. There were only three farms whose owners did not engage in the activities, leaving it to their employees to participate in the project. A general overview of the farm was obtained in the first extension activity of “Walk around the farm.” These walks revealed that the pig sheds varied from simple and damaged constructions, as in farms A, C, E and J, to slightly more sophisticated masonry buildings, equipped with pig nipple drinkers, as well as the inclusion of materials for entertainment and environmental enrichment found at farms D, F and I.

The proposal of the field day, i.e., monitoring the cleaning of the facilities by the farmer with the members of the team, revealed that the farmers cleaned the pig sheds using only water and a broom, except on farm J, which had straw litter covering the bare floor. On the field day, only farms F and I cleaned the pig sheds using pressurized water.

Farm residents and employees, including those that did not participate in the fecal parasite survey, took active part in the interactive lecture, which was attended by 34 people. The pictures in the booklet used in the lecture clearly attracted the participants’ attention. In the “happy pig and sad pig” activity carried out that same day, the most frequent incorrect handling behavior was washing the pig sheds using only water and a broom, and this occurred at all the farms except at farm J. Moreover, at eight of the ten farms (A–D, F, H–J), the people that took care of the pigs did not wear specific work clothes solely to handle the animals, and at seven of the 10 farms (B–G, I), the feces and food scraps were not removed dry from the pig sheds. Other mishandling practices observed by the team and pointed out in this extension activity were the presence of animals in dirty, wet or muddy sheds, which was observed at farms B, C, E and G; failure to wash the drinkers and feeders daily, which was found at farms C, D, F–H; and feeding the pigs with smelly spoiled food, which the team at farms A–C and G smelled and saw.

**Figure 2:**
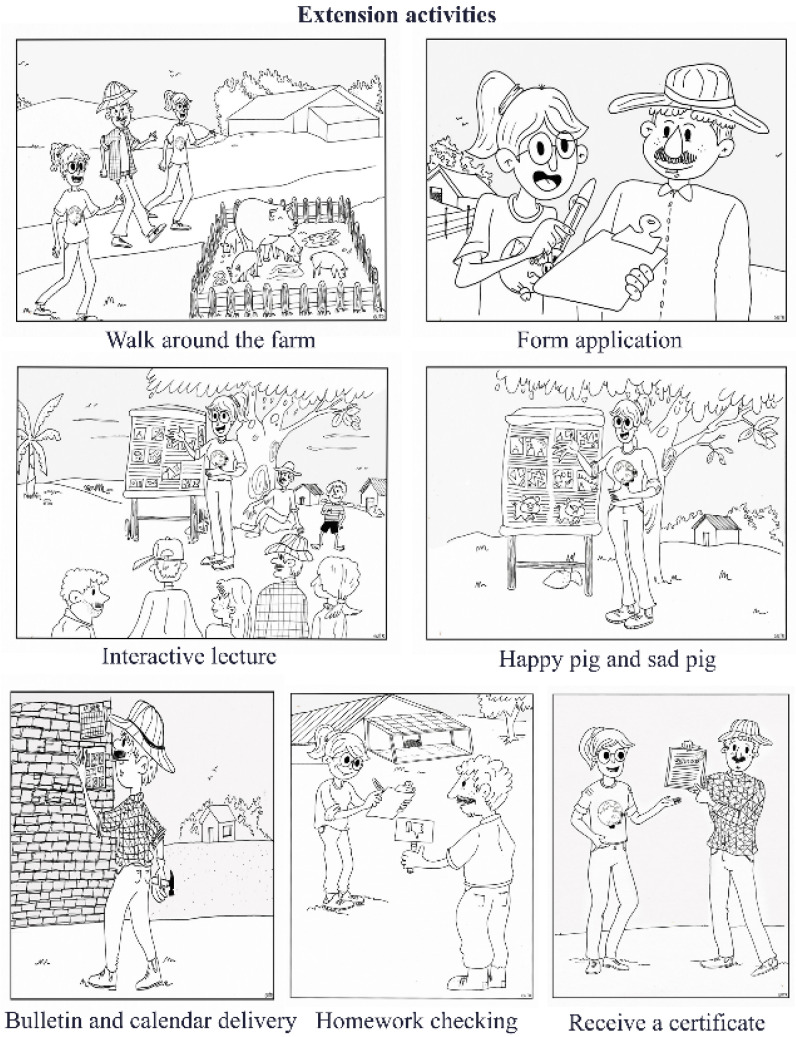
Some extension activities applied on family farms for raising pigs.

During the field work, several participants of the study at half the farms (C, D, E, F and I) were seen nailing the bulletins and calendars provided by the research team onto shed walls. In addition, the pigs at all the farms were treated with anthelmintics via their food or water in the presence of the research team members. The “homework-checking” activity, whose purpose was to determine whether the information provided in previous visits had been retained, was answered interactively with 100% of correct answers by eight pig caretakers from farms B to I. People from the different pig family farms that took part in this study were happy to receive the certificate of participation (Figure 2).

## 3. Discussion

In the state of Rio de Janeiro, these pig farms are usually concentrated in areas further away from the large urban center. In this regard, the municipality of Cachoeiras de Macacu stands out in the state because of its long-standing reputation for breeding and raising livestock. Despite this status, few scientific studies of these animals have been conducted in this municipality, including parasitological surveys. To make up for this lack of information, 10 family pig farms volunteered to participate in this study, leading to the detection of 86.1% of fecal parasites from pigs raised on family-run farms, with positivity rates similar or higher than the general rates found in most pig herds.

Lower intestinal parasite infection rates than those in this study have been detected in pig feces from farms located in other municipalities in Brazil, such as in the semi-confined production system in Mossoró, Rio Grande do Norte and in Pinheral, Rio de Janeiro, in which 72.7% and 30% of positivity rates, respectively, were found, as well as at family farms in Paraíba (79.5%), Rio Grande do Sul (43.2%) and Minas Gerais (62.9%) [18,19,20,21,22]. Case records of parasite infections in pigs lower than those in this study, varying from 13.2% to 84.6%, have also been reported on family farms in other countries, including Peru, Venezuela, Ethiopia, Uganda, Rwanda and India [16,23,24,25,26,27].

Lower frequencies for gastrointestinal parasites in pig in industrial systems in Brazil type were in other cities in the state of Rio de Janeiro, Minas Gerais and São Paulo, ranging from 38.6% to 59.1% [13,28]. Lower frequency of parasites was also evidenced in industrial properties located in other countries such as China, Korea and France [29,30,31].

Parasite infection rates higher than those of this fecal parasite survey have been reported on farms located in other municipalities of the state of Rio de Janeiro and in Sergipe, Brazil, ranging from 88% to 100%, as well as a family-run pig farms in Colombia (91%) and Nigeria (86.6%) [13,17,32,33,34,35].

The different frequencies mentioned above among the studies may be directly related to differences in the sample size, to the different laboratory tests used, the geographical location of the property and mainly to the handling performed with the animals. The high positivity rate found on family pig farms in Rio de Janeiro was actually expected, since this problem had already been reported in other studies in Brazil. Although the pigs in these family farms are kept in confinement, few producers have invested in the sanitary management of their herd. This problem was revealed by the answers to the questionnaires as well as in the field activities. None of the farmers reported using detergents and/or disinfectants to clean the pig sheds, and none of them used a sanitary break or a flamethrower to sanitize the facilities. These findings also apply to subsistence pig farms in Pernambuco, Tanguá, Rio de Janeiro, Brazil as well as to small pig farms in Sweden [17,36,37].

All the pig farm owners and/or caretakers included in this study reported habitually hosing down the animals, regardless of the environmental temperature on any given day, leaving a high moisture content in the sheds and the pigs’ bodies soaked in water. This situation was also observed by team members during the extension activities of “Walk around the farm” and “Field day.” Although all the farmers stated that they treated their pigs with anthelmintics, they had no specific deworming schedule for their animals. This problematic situation may have favored the survival of evolutionary forms of parasites in the animals’ enclosures as well as reinfections.

Although slightly more protozoa than helminths were detected, both case records exceeded 70%, indicating an irregular application of antiparasitic drugs. Moreover, it should be noted that most of the antiparasitic drugs administered to animals mainly eliminate helminths, being considered only anthelmintics. Drugs that aim to control infection by coccidia are also generally administered to piglets on industrial farms in Brazil [38].

The most frequently detected parasites in pig feces were cysts and trophozoites of the phylum Ciliophora similar to *Balantioides coli*. The phylum Ciliophora was identified in both sexes, particularly in the growing-finishing age groups, which are directly associated with protozoan infections. Similar situations have been reported at family farms in other municipalities in the state of Rio de Janeiro, Brazil, as well as in Venezuela [13,17,23]. The high positivity rate of this protozoan may be directly associated with the animals’ diet, which is generally composed of leftovers of human food mixed with wheat bran or various types of commercial hog feed. The inclusion of human food leftovers fed to pigs has also been reported at hog farms in other Brazilian states as well as in countries such as Ethiopia, Cameroon, Uganda and Colombia [3,8,17,25,35,39,40,41].

Wheat is known to be rich in carbohydrates, which is a nutrient that serves as the main energy source of protozoa such as *Balantioides coli*, especially when it is kept in vitro [13,42]. In this study, it was found that a carbohydrate-rich diet such as wheat generally represented one of the main components of the animals’ diet and was present in all the analyzed food types, which may have masked its significant relevance in the records of this protozoan.

Pigs are considered the main reservoirs of *B.* *coli,* and this parasite is frequently detected in the feces of these animals. However, the confirmation of this species can only be performed using molecular techniques, since this ciliate is morphologically similar to other species such as *Buxtonella* sp. Infections in humans with *B. coli* usually occur in rural areas where pigs are raised [43]. However, in the present study, forms compatible with that of this protozoan were not detected in stool samples from farmer owners, pig caretakers and other family members. In addition, most of the participants stated in the questionnaire that they had never seen blood in their stools, which is an important finding in terms of human balantidiasis, since dysentery, i.e., diarrhea stools with mucus and blood, is considered the main symptom of this infection [43]. A similar situation, i.e., a high frequency of forms compatible with *B. coli* in pig feces and their absence in the stool of pig farmers, has been reported in communities in Venezuela and in other municipalities in the metropolitan area of Rio de Janeiro [17,44,45].

In addition to protozoan forms compatible with *B. coli*, non-sporulating coccidian oocysts, amoebic and *Blastocystis* sp. cysts have also been detected. Coccidian oocysts have frequently been found in the feces of pigs raised on family farms and commercial hog farms in several Brazilian states, such as Bahia, in other municipalities of the metropolitan area and in Vale do Paraíba in the states of Rio de Janeiro, Maranhão and Paraíba [13,17,20,21,32,46,47]. The pathogenesis with clinical signs of coccidiosis is usually observed in suckling piglets infected with *Cystoisospora suis* [48]. Such oocysts were detected in all the age groups in this study. However, they were not detected in pig feces from farms B and F, where the only pigs were in the finishing phase, which is the period when they are the most resistant to infections. Cysts of uninucleate amoeboids, including *Entamoeba polecki* and *Entamoeba suis*, which have been found infecting pigs, as well as *Blastocystis* sp., do not seem to have negative impacts on pig production. However, we found no studies correlating these infections with clinical cases among pigs. Nevertheless, their zoonotic potential should be highlighted, especially that of *E. polecki* and of *Blastocystis* sp. [14]. Although they were detected, evolutionary forms of amoeboids and *Blastocystis* sp. were not recovered from the feces of pigs raised at all the farms in this study, and their presence was low compared to that of other parasites.

It should be noted that the diagnosis of protozoa, especially coccidia, in the feces of the animals was expected, because even though the caretakers stated they treated the animals with anthelmintics, these drugs are known to be ineffective in eliminating protozoan infections. Moreover, it must be kept in mind that infectious agents must be kept under control in the facilities where the animals are housed, since the structures of oocysts and cysts are highly resistant to chemicals and different temperatures. Poor environmental hygiene was common at most of the family farms in this study. Hence, it can be inferred that the main facilitating factor for reinfections by these agents may be contaminated facilities. In this regard, what stood out was the presence of coccidia, which were detected at the highest rate in the pigs at farm J, whose pens were not cleaned because they had packed earth floors covered with straw bedding. Added to this incorrect practice is the high-carbohydrate diet the pigs are fed and the poor care given to newborn piglets, such as inappropriate management practices that potentiate infections by protozoa, particularly by coccidia.

The helminths most detected in pig feces were strongylids, which were followed by *Strongyloides ransomi*. Strongylids were was proportionally more detected in the older age groups, including finishing, which was followed by the growing phase. These age groups were significantly associated with infection by this nematode, remaining in this condition even in the multivariate analysis. On the other hand, *S. ransomi* was detected mainly in the feces of females. In the logistic regression model, the sex of the pigs was not significantly associated with the presence of this parasite. This situation was already expected, since the frequency of this parasite is not related to the sex of the animal but to the age group, which is a variable that also did not show a significant difference in the present study.

Note that in this study, eggs of nematodes possibly belonging to the superfamilies *Strongyloidea* and *Trichostrongyloidea* were included in the category of strongylids. Eggs in this group are identical, requiring more sophisticated laboratory techniques to distinguish the species, such as fecal culture [49] or molecular biology. It is noteworthy that biological samples in which larval eggs of *S. ransomi* were detected simultaneously contained nematode larvae, suggesting that they also belong to this same parasite taxon. It is known that the eggs of this nematode embryonate very quickly, starting in the host’s intestines. Therefore, our priority was to process the fecal samples in the laboratory soon after they were collected, thereby increasing the probability of recovering eggs and this facilitating the diagnosis of this parasite.

In the case of strongylids, the larvae of these nematodes can emerge from hypobiosis in the stomach and intestinal mucosa of sows close to parturition, stimulated by hormonal changes, and consequently become adult parasites during the period in which the sows are suckling their litter, which is when their energy expenditure is at the highest level. Thus, the infection of sows by strongylids, that is, of older females, can lead to the lean sow syndrome, which can culminate in death [49].

Although this is not a clinical study, several farms were found to have breeding sows with sub-optimal body scores and piglets suffering from dwarfism. It is also important to highlight, once again, that the carbohydrate-rich diet and negligent care provided to newborn piglets, as reported by the producers, proved to be a risk factor for *S. ransomi*. In fact, the main practice reported in the care of piglets was allowing newborns to suckle immediately after birth. Although suckling is extremely relevant for the animals, it may have favored the transmammary transmission of the parasite, since pregnant sows were not dewormed in the weeks preceding parturition. Another relevant risk factor for infections by strongylids and *S. ransomi* is the poor hygiene of the facilities found in this study and the lack of recommended washing of the pig pens at farm J. In addition, washing the concrete floors of pig barns solely with water may have been a factor of confusion on the family farms of this study with respect to the frequency of strongylids since, if not properly sanitized, concrete floors and walls offer suitable conditions for the maintenance of the infective larvae of this parasite.

Other nematodes were also detected in most of the pig herds in this study, including *Ascaris suum* and *Trichuris suis*. In fact, the former nematode of *Ascaris suum* was even detected in its adult form, which was being eliminated by the pigs in the presence of the team during their technical visits. Surprisingly, the presence of *A. suum* and *T. suis* identified in the feces of pigs from Cachoeiras de Macacu, RJ was higher than that recovered in most fecal parasite surveys carried out in Brazil [13,17,20,32,35,46,50].

Higher infection rates than those in this study have been reported only at family farms in Rio Grande do Sul and Paraíba, i.e., 43.2% of *A. suum* in the former state and 30.2% if *T. suis* in the latter [21,22]. These nematodes are highly relevant in pig farming, because they cause reduced weight gains in the fattening phase, i.e., in the growing-finishing phase, since their pre-patent period varies by about eight weeks [49]. The presence of the nematodes identified in the feces of the animals indicates that pig facilities favor the development of infective parasitic forms, such as the geohelminths *A. suum*, *T. suis*, strongylids and *S. ransomi*.

A remarkable situation in Cachoeiras de Macacu, RJ, which may have favored the widespread presence of geohelminths and other parasites in general, was the conditions evidenced in the pig sheds, particularly the high levels of moisture, i.e., wet floors and water puddles. This situation was the result of hosing down the pig barns with water, in many cases river or spring water. This moisture, allied to the tropical humid climate of Rio de Janeiro, provides an optimal environment for the development of infectious parasites. This factor, combined with pig barns partially built of wood, as seen at farms A and B, which facilitates the retention of organic matter and moisture, stood out as risk factors for the maintenance of ascariasis at the pig farms analyzed in this study. In addition, pig feeders and waterers set directly upon the floor favor the contamination of water and food with the feces of animals in the pigsty, predisposing them to reinfections. Unfortunately, although pig nipple drinkers prevent this problem of contamination, they were found only at three farms, D, F and I, and only Farm I had suspended trough feeders in the piglets’ pens. The erroneous management practices associated with the climatic conditions of the place may have contributed jointly to the frequency of some parasites, highlighting those that have infective forms of high environmental resistance, such as the eggs of *Ascaris suum* and *Trichuris suis*. Thus, despite the sampling used in the present study being above the recommended level to reach the level of significance, it may still not have been adequate to extrapolate the relevance of some variables in relation to these parasites. Since several mismanagement behaviors observed in the farms may have contributed to the fecal oral transmission of the larvae eggs of these parasites.

In addition to the aforementioned parasites, a *Capillaria* sp. egg was also detected in the feces of a pig. It should be noted that this nematode does not usually infect pig; therefore, its detection may be associated with cases of pseudoparasitism. It is possible that the pig ingested the *Capillaria* sp. egg through the feces of other animals or the predation of synanthropic animals, such as rodents. In this study, almost all the farmers reported the presence of rodents in the peridomicile and stated they use chemical products for their control, corroborating a case of pseudoparasitism. This sanitary problem has also been encountered at pig farms in France and in the municipality of Tanguá, Rio de Janeiro [17,31].

Despite the high frequency of gastrointestinal parasites detected in pig feces, the stool samples from farmers and their families showed a low parasite positivity rate. This fact may be attributed directly to the experimental period of this parasitological survey, which was conducted during the coronavirus pandemic, and the excessive use of antiparasitic drugs such as ivermectin for the treatment and prophylaxis of coronavirus infection by the population. During the application of the questionnaire and the extension activity that consisted of an interactive lecture with a book entitled “Parasites and the importance of their control,” several participants reported having used ivermectin on more than one occasion to avoid infection by the virus, although most of them were unfamiliar with the anthelmintic function of this drug. This drug was being taken incorrectly because numerous public authorities and politicians in Brazil, notwithstanding their scientific illiteracy, defended the use of antiparasitic drugs such as ivermectin for the prophylaxis and treatment of COVID-19 infection and encouraged it on social media. This excessive and erroneous use of antiparasitic drugs can reduce the frequency of parasites in the population but may, in the future, stimulate parasite resistance to these drugs.

In addition to the investigation of intestinal parasites, mainly *Sarcoptes scabiei* var. *suis*, skin scrapings from the pigs’ ears were also analyzed to check for arthropods. In this investigation, evolutionary forms of this parasite were detected in 3.5% of the pigs, mainly in biological samples of animals from the finishing group. However, studies conducted at other pig farms in Brazil have reported the detection of higher infestation rates by this arthropod (12.1% to 43%) [17,51,52]. Sarcoptic mange can be a problem in pig raising and may reduce the animal production performance, given that pigs infested by the mite may suffer from severe itching, causing them to stop feeding and thus reduce their feed conversion efficiency [53]. It is worth noting that this infestation rate might have been even higher if deeper skin scrapings had been taken from the epidermis of the ear pinna, reaching the intradermal galleries where most mites are lodged as well as other parts of the animals’ bodies. Despite the low sensitivity of the laboratory technique, the identification of the mite, albeit at low rates, underscores the precariousness of sanitation management, particularly the absence of a quarantine period for newly purchased animals as well as the overcrowding of pig stalls, since the mite is transmitted mainly via direct contact between animals.

It is worth mentioning that one of the limiting factors of this study was the non-identification of some parasite taxa in a more specific way due to the non-use of laboratory techniques such as sporulation of oocysts and molecular biology. Furthermore, if these had been used, perhaps the parasite frequency would have been even higher. This panorama highlights the need for investment by research agencies in Brazil that enrich the literature with epidemiological data.

Among the extension proposals used, the “Walk around the farm” provided a view of the differences between the farms, and an initial diagnosis of problems encountered in pig raising, particularly insofar as they concern the facilities, as well as talking with the farmers and listening to what they had to say. Walking around a farm is helpful to reach a participatory diagnosis and to garner information used in plans for later application in rural areas [54]. After these initial impressions, the activity of mediation of information through the interactive lecture with an instrument similar to a large book was well attended by the participants, especially when the information under discussion was illustrated with real pictures or photographs. The strategy of using pictures, especially real ones such as photographs, facilitates the transmission of information in field extension activities, especially for people with low levels of literacy [17].

Pictures in the form of drawings were also used in this study in the “Happy pig and sad pig” activity, which was deemed a success, since the farmers themselves decided whether or not their behavior was suitable and if it should be illustrated by a happy pig or a sad one. The farmers interacted dynamically in this activity, which was characterized by a relaxed and unconstrained atmosphere. This activity enabled us to remind everyone about the main incorrect pig-handling practices detected on the “Field day” and go over the information garnered from the questionnaire and from the walk around the farm, highlighting as erroneous conduct the cleaning the pig pens only with water and a broom. At the end of the visits, the farmers stated their satisfaction in receiving free anthelmintics for the pigs and a bulletin and calendar to remind them about important points in pig management practices and to encourage them to treat their animals with medications such as antiparasitic drugs.

Unfortunately, much of the information passed on through extension activities may be lost over time if continuous actions are not taken [17]. However, on the last visit, a question-and-answer game called “Homework checking” indicated that the farmers still remembered the information that had been imparted to them during the research team’s visits to the farms. Upon the conclusion of all the activities, the farmers and their family members were given a certificate of participation, whereupon they clearly expressed their satisfaction in participating in the study.

It is important to note that this study combined scientific research within the scope of a parasite survey and a qualitative assessment based on a questionnaire to identify risk factors and extension activities, aiming to provide family pig farmers with immediate feedback about the research findings and useful information to help them minimize the transmission of parasites, including those with zoonotic potential. Studies combining scientific research and extension activities with family pig farmers are practically non-existent. Only one article published by our group was found in the literature [17], highlighting the pioneering and importance of this type of study.

## 4. Materials and Methods

### 4.1. Study Location

This study was carried out between December 2020 and August 2021 on family-owned pig farms located in the municipality of Cachoeiras de Macacu, which covers an area of 954,749 km^2^ and is part of the metropolitan area of the state of Rio de Janeiro. This municipality shares borders with the municipalities of Nova Friburgo, Silva Jardim, Rio Bonito, Tanguá, Itaboraí, Guapimirim and Teresópolis. Moreover, Cachoeiras de Macacu is divided into three districts: the First District, Japuíba and Subaio, and it has a population of about 54,273 [55].

This municipality has a wide variety of water sources and is a major supplier of water to other locations. The municipality has an extensive area dedicated to agricultural activity as well as large areas that are unoccupied or whose land is unplanned, and its local agricultural sector is based on family farming [56]. This family-based agricultural sector has long been organized into associations and cooperatives. In addition, currently in the city of Cachoeiras de Macacu, there is no industrial type property for raising pigs.

Due to a lack of information on the amount of family farms in Cachoeiras de Macacu city, the sample calculation to reach a confidence level of 95% was based on other parasitological surveys carried out with pig from family properties in other cities in Rio de Janeiro state, which detected frequencies ranging from 88.6% to 93.1% [13,17].

The properties known to the research team were invited, and they shared information about this study with other family farms. At the end, the family-run pig farms involved in this study are identified here by the letters A, B, C, D, E, F, G, H, I and J to preserve their anonymity.

### 4.2. Study Design and Collection of Biological Samples

In this study, three technical visits were made to each farm for various purposes. The visits took place at intervals of 4 to 7 days.

First visit: Consisted of presenting the study in a simple conversation with farm owners and other family members and obtaining their signature on the informed consent forms required by the Ethics Committees. The farmers that agreed to participate in the study filled out two semi-structured forms: the first containing questions about the pigs and pig-raising management, which was answered only by the producer, and the second containing sanitation-related questions, which was answered individually by each family member. After the forms were filled out, an extension activity of a “Walk around the farm” was carried out to familiarize the researchers with the property and the pig-raising operation. Fecal samples were collected directly from the rectal ampulla of pigs using a rectal palpation glove lubricated with glycerin. Material was scraped from the outer ears using a stainless-steel spatula soaked in glycerin and was deposited on sterile plastic Petri dishes, which were sealed with tape. In addition, during this visit, stool collection kits were delivered to farmers and their family members for their own use. These kits contained two labeled 80 mL stool specimen containers, one with and the other without chemical preservative, two wooden spatulas and a leaflet describing the proper collection procedure. Each person was also told to collect a sample on two different days and to store them in a refrigerator. The stool samples from each person were picked up by the study team at a prearranged time.

Second visit: Included the delivery of human and pig parasite test results. The pig test results were explained to each farmer, and the human test results were explained to each individual to clear up any doubts. An extension activity called “Field Day” was carried out during this second visit. This activity consisted of accompanying the farmer on his daily routine activities of cleaning pig pens and sanitizing the animal feed and water troughs. In addition, a presentation entitled “Parasites and the importance of their control” was given during this visit, using a structure resembling a large book made of canvas posters containing pictures and “Happy pig and sad pig” activities. This activity was carried out on a poster containing 10 sentences associated with drawings of pig-handling activities. If the producer stated that the handling was performed correctly, it was given the picture of a happy pig, but if the behavior was inadequate, it was given the image of a sad pig. At the end of this visit, the producer was given a bulletin describing ten important steps for the producer on proper hygiene in pig farming and a health calendar for scheduling medications and reminding the farmer of the dates and names of drugs to administer to their pigs (Figure 2).

Third visit: During this visit, antiparasitic drugs containing fenbendazole as active ingredient were mixed into the pig feed or water. In addition, the team engaged in an activity called “Homework checking,” which involved an interactive game of questions and answers to determine, by means of 10 sentences, if the information provided to the farmer was clearly understood. At the end of this visit, the participants of this study received a printed certificate of participation (Figure 2).

### 4.3. Laboratory Processing

The human and pig fecal samples were immediately processed by direct examination. At the examination time, each fecal sample was sub-sampled, taking new samples both from the surface and a more profound point. The fecal material from this scraping was homogenized in a sterile buffered saline solution. An aliquot of this fecal solution was analyzed under an optical microscope.

Another part of the sample was homogenized and filtered. The filtrate was aliquoted in 15 mL centrifuge tubes with a conical bottom to carry out the centrifugal sedimentation techniques (Ritchie-modified by Young et al. [57,58]) and centrifugal flotation (Sheather-modified by Huber et al. [59,60]). In the centrifugal sedimentation (Ritchie-modified), 3 mL of ethyl acetate with a drop of neutral detergent was added to the 7 mL fecal solution, and then, it was centrifuged at 2000 rpm for 2 min. The supernatant was discarded, and the sediment was resuspended in distilled water to a volume of 7 mL, repeating the centrifugation step. Finally, the pellet was transferred to a microscope slide covered with a coverslip. In the centrifugal flotation (Sheather-modified), the fecal solution was centrifuged at 1500 rpm for 10 min. Then, the supernatant was discarded, and a sucrose solution with a density of 1300 g/mL was added, repeating the centrifugation at 1500 rpm for 5 min. A new sucrose solution was added until a positive meniscus was formed; a coverslip was placed over it and left to rest for 4 min. In the end, this coverslip was placed on a slide and observed under a microscope.

In addition, part of the filtered material was left to sediment in conical bottom glasses over 24 h to perform Lutz’s spontaneous sedimentation technique [61]. Moreover, the Petri dishes containing the ear-scrap samples were processed and examined in an Olympus^®^ CKX41 inverted microscope under 100× magnification [17]. The Petri dishes were then covered with a lactophenol solution (percentage or concentration of the lactophenol needed here), sealed, and incubated at room temperature for one month. After this period, 1 mL of the sediment was transferred to a centrifuge tube, adding distilled water to complete a final volume of 15 mL, and centrifuging at 2500 rpm for 5 min. The supernatant was discarded, and a sample of the pellet was put on a microscope slide and examined under an optical microscope. After looking at the slide, the remaining pellet was suspended with a sucrose solution at a density of 1,300g/mL to a final volume of 15 mL. This solution was then centrifuged at 1500 rpm for 10 min. After this procedure, the tubes were placed on shelves, and sucrose solution was added until a meniscus was formed. On top of the meniscus, coverslips were placed and left to rest for 20 min, and then, they were placed on a slide and observed under a microscope.

One microscope slide of each technique was generated. An Olympus BX 41 optical microscope was used to examine the slides prepared by each technique and to produce photographic documentation, initially under 50× and 100× magnification, and then under 400× for confirmation, when necessary.

### 4.4. Data Analysis

A biological sample was considered positive when at least one evolutionary form of parasites was found (trophozoites, eggs, larvae, cysts or oocysts). The frequency was determined by dividing the number of positive samples by the total number of samples collected, and these data were presented in percentages (%). All the qualitative information retrieved from the forms was tabulated and presented descriptively, by means of a percentage, while the most widely reported information was presented in tables. Data on the animals’ sex and age, in months, were also retrieved and tabulated. Thus, the pigs were classified in stages as follows: Initial stage—from one to two months of age to weaners, Growing stage—from two to four months old, and Fattening stage—four months and older.

All the data retrieved from the forms and the information about the animals’ sex and age were stored in Excel Microsoft Office 2007 database templates. Statistical analyses were performed to determine the significance of the frequency of parasites among the family farms and to ascertain if there was any significant association between the information obtained from the forms about the animals’ sex and age and parasite positivity. A univariate exploratory data analysis was initially performed to select variables with *p* ≤ 0.05 based on the chi-square test or Fisher’s exact test. This was followed by a multivariate logistic regression analysis of the significant variables, at a 5% level of significance, in which possible risk factors were analyzed using the Odds Ratio (OR) and their respective 95% confidence intervals. All the statistical analyses were performed using Epi Info^TM^ software.

## 5. Conclusions

The prevalence rate of parasites in pigs at these farms was high, particularly gastrointestinal ones, and this may lead not only to financial losses for the owners but also to problems of animal welfare. This problem was an indication of how these small farmers are neglected by public authorities given that they need these animals as a source of food protein, especially during the current COVID pandemic, when the price of beef and the impoverishment of the Brazilian population have increased dramatically. It should be pointed out that the high parasite positivity rate found among the pigs in this study seems to be directly attributable to the poor sanitary practices adopted by the breeders of these animals, which was revealed not only through information garnered from the questionnaire but also through the research team’s observations during their technical visits to the farms. However, it is evident that these family farmers lack the financial and technical conditions needed for the proper handling of their animals. Moreover, they are not given suitable and necessary information that would enable them to improve their production in terms of animal health.

## Figures and Tables

**Table 1:** Parasites detected in fecal samples and ear scrapings from pigs raised on different family farms.

Parasites	Family Pig-Farming Properties											
	A (*n* = 36)	B (*n* = 3)	C (*n* = 6)	D (*n* = 27)	E (*n* = 8)	F (*n* = 4)	G (*n* = 15)	H (*n* = 28)	I (*n* = 41)	J (*n* = 12)	Total (*n* = 180)	*p* Value
Phylum Ciliophora	19 (52.8%)	2 (66.7%)	1 (16.7%)	25 (92.6%)	7 (87.5%)	3 (75%)	14 (93.3%)	25 (89.3%)	20 (48.8%)	11 (91.7%)	127 (70.5%)	0.00 *
Coccidia oocysts	16 (44.4%)	0	1 (16.7%)	10 (37%)	5 (62.5%)	0	10 (66.7%)	13 (46.4%)	4 (9.7%)	10 (83.3%)	69 (38.3%)	0.00 *
Amoebids	1 (2.8%)	0	0	2 (7.4%)	0	0	1 (6.7%)	1 (3.6%)	0	0	5 (2.8%)	1
*Blastocystis* sp.	0	0	0	0	2 (25%)	0	0	2 (7.1%)	5 (12.2%)	0	7 (3.9%)	0.61
*A. suum*	24 (66.7%)	3 (100%)	4 (66.7%)	0	3 (37.5%)	0	2 (13.3%)	16 (57.15%)	2 (4.9%)	4 (33.3%)	58 (32.2%)	0.00 *
*T. suis*	6 (16.7%)	2 (66.7%)	0	8 (29.6%)	2 (25%)	0	2 (13.3%)	3 (10.7%)	0	8 (66.7%)	31 (17.2%)	0.009 *
Strongyles	19 (52.7%)	1 (33.3%)	5 (83.3%)	19 (70.4%)	8 (100%)	4 (100%)	10 (66.7%)	24 (89.3%)	1 (2.4%)	11 (91.7%)	102 (56.7%)	0.00 *
*S. ransomi*	18 (50%)	1 (33.3%)	3 (50%)	16 (59.2%)	5 (63.5%)	0	6 (40%)	20 (71.4%)	0	11 (91.7%)	80 (44.4%)	0.00 *
Nematode larvae	2 (5.5%)	0	1 (16.7%)	0	1 (12.5%)	0	0	1 (3.5%)	0	2 (16.7%)	7 (3.9%)	0.59
*Capillaria* sp.	1 (2.8%)	0	0	0	0	0	0	0	0	0	1 (0.5%)	1
**Subtotal**	32 (88.9%)	3 (100%)	6 (100%)	25 (92.6%)	8 (100%)	4 (100%)	14 (93.3%)	27 (96.4%)	25 (61%)	11 (91.7%)	155 (86.1%)	
**Ectoparasites**	**A ** **(*n* = 35)**	**B** **(*n* = 3)**	**C** **(*n* = 5)**	**D** **(*n* = 19)**	**E** **(*n* = 8)**	**F** **(*n* = 3)**	**G** **(*n* = 7)**	**H** **(*n* = 21)**	**I** **(*n* = 32)**	**J** **(*n* = 9)**	**Total ** **(*n* = 142)**	***p* Value**
*S. scabiei* var. *suis*	0	0	2 (40%)	0	1 (12.5%)	0	1 (14.3%)	1 (4.8%)	0	0	5 (3.5%)	1
*D. phylloides*	1 (2.9%)	0	0	0	0	0	0	0	0	0	1 (0.7%)	1
Arthropod egg	0	0	1 (20%)	0	3 (37.5%)	0	2 (28.6%)	1 (4.8%)	2 (6.2%)	0	9 (6.3%)	1
**Subtotal**	1 (2.8%)	0	2 (4%)	0	4 (50%)	0	2 (28.6%)	2 (9.5%)	2 (6.2%)	0	13 (9.1%)	

Family pig farms identified by the letters A to J; * *p*-value ≤ 0.05. Strongylids: Eggs of the superfamilies *Trichostrongyloidea* and *Strongyloidea*.

**Table 2:** Univariate analysis of general information about gastrointestinal parasites detected in fecal samples from pigs raised on family farms.

Information	Parasite		Phylum Ciliophora		Coccidia Oocysts		*A. suum*		*T. suis*		Strongyles		*S. ransomi*	
	%	*p* Value	%	*p* Value	%	*p* Value	%	*p* Value	%	*p* Value	%	*p* Value	%	*p* Value
**Sex**														
Male (*n* = 71)	84.5	0.6623b	67.6	0.5067b	35.2	0.5324b	25.3	0.1419b	16.9	1b	47.9	0.06b	35.2	0.0476b *
Female (*n* = 109)	87.1		72.5		40.4		36.7		19.4		62.4		50.4	
**Age range**														
Initiation (*n* = 79)	72.1	0.0000a *	55.7	0.005a *	39.2	0.4986a	27.8	0.1924a	24	0.0616a	40.5	0.006a *	38	0.3026a
Growing (*n* = 28)	100		85.7		28.6		46.4		17.9		67.8		50	
Fatteners (*n* = 73)	95.9		80.8		41.1		86.3		9.6		69.9		49.3	
**Property type**														
Backyard of the homes (*n* = 47)	91.5	0.3257b	59.6	0.06b *	44.6	1b	63.8	0.0000b *	21.3	1b	59.6	0.1472b	51.06	0.6305b
Little farm (*n* = 133)	84.2		74.4		36.1		21.1		15.7		55.6		42.1	
**Stocking and categorization of animals by pig pen**														
There is no set number (*n* = 99)	92.9	0.0058a *	76.8	0.111a	54.5	0.000a *	49.5	0.0000a *	21.2	0.2338a	72.7	0.0000a *	60.1	0.0000a *
Divide pigs by age group (*n* = 77)	76.6		62.3		19.5		11.7		12.9		36.7		25.9	
Only one pig per pen (*n* = 4)	100		75		0		0		0		100		0	
**Facilities type**														
Collective bays with cement wall (*n* = 100)	81	0.06a	73	0.0238a *	27	0.003a *	18	0.0000a *	11	0.0275a *	48	0.0005a *	36	0.0211a *
Collective bays with cement wall, wooden or bamboo fence (*n* = 41)	95.1		80.5		63.4		31.7		29.3		82.9		60.9	
Collective bays with cement and wood wall (*n* = 39)	89.7		70.6		41.03		69		20.5		51.3		48.7	
**Roof pen**														
Totally covered with fiber cement tile, galvanized or PVC (*n* = 168)	85.7	1b	69	0.1140b	35.1	0.001b *	32.1	1b	13.7	0.0001b *	54.1	0.0135b *	41.1	0.0006b *
Partially covered with fiber cement tile (*n* = 12)	91.7		91.7		83.3		33.3		66.7		91.6		91.7	
**Floor of the buildings**														
Naked soil or deteriorated cement (*n* = 33)	93.4	0.262b	78.8	0.2958b	63.6	0.001b *	30.3	0.8399b	30.3	0.0397b *	78.8	0.005b *	60.6	0.051b
Cemented or concreted (*n* = 147)	84.4		68.7		32.6		32.7		14.3		51.7		40.8	
**Water to cool the pig**														
Yes (*n* = 36)	88.9	0.788b	52.8	0.0134b *	44.4	0.445b	66.7	0.0000b *	16.7	1b	52.7	0.7073b	50	0.4056b
No (*n* = 144)	85.4		75		36.8		23.7		17.4		57.6		43.06	
**Habit of throwing water on the body of pigs to refresh them**														
Yes (*n* = 180)	86.1	NA	70.5	NA	38.3	NA	32.2	NA	17.2	NA	56.7	NA	44.4	NA
**Supply of drinking water**														
* Ad libitum* (*n* = 159)	84.9	0.316b	71	0.7992b	36.5	0.231b	29.6	0.0467b *	13.2	0.0005b *	53.5	0.019b *	40.8	0.100b
Provided two or three times a day (*n* = 21)	95.2		66.7		52.4		52.4		47.6		80.9		71.4	
**Type of drinking fountains**														
Cement fountain (*n* = 52)	96.15	0.004a *	80.8	0.1250a	46.1	0.0002a *	48.1	0.0000a *	13.5	0.0564a	76.9	0.000a *	57.7	0.0000a *
Nipple type (*n* = 72)	75		66.7		19.4		2.7		11.1		33.3		22.2	
Cut tire and plastic bowls (*n* = 8)	100		87.5		62.5		37.5		25		100		62.5	
Cement lame and plastic bowls (48)	89.6		62.5		54.2		58.3		29.2		62.5		60.4	
**Food provided to pig**														
Remains of human and agricultural food, wheat bran and/or barley (*n* = 63)	95.2	0.000a *	90.5	0.000a *	60.3	0.0000a *	39.7	0.0000a *	23.8	0.0067a *	84.1	0.0000a *	66.7	0.0000a *
Remains of human and agricultural food, maize or rice flour (*n* = 45)	91.1		48.9		37.8		68.9		17.8		55.6		48.9	
Agricultural remainder and specific pig feed and wheat bran (*n* = 41)	60.9		48.8		9.8		4.9		0		2.4		0	
Horse feed, wheat bran, corn flour or rice and corn (*n* = 27)	92.6		92.6		37		0		29.6		70.4		59.3	
Corn bran for pig (*n* = 4)	100		75		0		0		0		100		0	
**Feeder**														
Cement feeder on the floor (*n* = 123)	83.7	0.462a	76.4	0.001a *	34.1	0.0022a *	18.7	0.0000a *	12.2	0.000a *	53.6	0.0710a	38.2	0.0042a *
Directly on the floor (*n* = 36)	88.9		52.8		44.4		66.7		16.7		52.8		50	
Floor and feeder (*n* = 9)	100		33.3		11.1		77.8		22.2		66.7		44.4	
Floor, plastic bowls and tire (*n* = 12)	91.7		91.7		83.3		33.3		66.7		91.7		91.7	

* *p* ≤ 0.05, a: Chi-square, b: Fisher’s exact test; NA: Not applicable as there are no two categories of answers.

**Table 3:** Univariate analysis of general and sanitary management about gastrointestinal parasites detected in fecal samples from pigs raised on family farms.

Information	Parasites		Phylum Ciliophora		Coccidia oocysts		*A. suum*		*Trichuris suis*		Strongylus		*S. ransomi*	
	%	*p* Value	%	*p* Value	%	*p* Value	%	*p* Value	%	*p* Value	%	*p* Value	%	*p* Value
**Presence of fly**														
Yes (*n* = 137)	82.5	0.010b *	65.7	0.0121b *	37.2	0.594b	26.3	0.0045b *	17.5	1b	47.4	0.0000b *	39.4	0.0216b *
No (*n* = 43)	97.7		86.1		41.9		51.2		16.3		86.1		60.5	
**Blood in pig feces**														
No (*n* = 180)	86.1	NA	70.5	NA	38.3	NA	32.2	NA	17.2	NA	56.7	NA	44.4	NA
**Anti-parasitic medicine**														
Yes (*n* = 180)	86.1	NA	70.5	NA	38.3	NA	32.2	NA	17.2	NA	56.7	NA	44.4	NA
**Caring for the piglets**														
Breastfeeding after birth and/or teeth cutting (*n* = 105)	93.3	0.0017b *	73.3	0.4071b *	52.4	0.000b *	50.5	0.0000b *	20	0.3173b	73.3	0.0000b *	60	0.0000b *
Breastfeeding after birth, vaccination, iron supplementation and sterilization (*n* = 75)	76		66.7		18.7		6.6		13.3		33.3		22.7	
**Accumulation of excreta in the pig enclosure**														
Yes (*n* = 31)	96.8	0.05b	75	0.6702b	50	0.1615b	37.5	0.5332b	18.7	0.7984b	75	0.0294b *	46.9	0.8451b
No (*n* = 148)	83.8		69.6		35.8		31.1		16.9		52.7		43.9	
**How to wash the environment**														
Water (*n* = 168)	85.7	1b	69.1	0.114b	35.1	0.0013b *	32.1	1b	13.7	0.0001b *	54.1	0.0135b *	41.1	0.0000b *
Not clean the environment, uses straw bedding (*n* = 12)	91.7		91.7		83.3		33.3		66.7		91.7		91.7	
**Sanitary break/Use of the flamethrower as a broom**														
No (*n* = 180)	86.1	NA	70.5	NA	38.3	NA	32.2	NA	17.2	NA	56.7	NA	44.4	NA
**Cleaning utensils intended only for cleaning the pig facility**														
Yes (*n* = 177)	85.8	1b	70.6	1b	38.9	0.2868b	31.07	0.0322b *	16.4	0.077b *	57.6	0.5796b	44.6	1b
No (*n* = 3)	100		66.7		0		100		66.7		33.3		33.3	
**Specific clothing only for handling pigs**														
Yes (*n* = 8)	100	0.332a	87.5	0.062a	62.5	0.017a *	37.5	0.2564a	25	0.779a	100	0.0251a *	62.5	0.5511a
No (*n* = 157)	84.7		67.5		34.4		33.7		17.02		53.5		43.9	
Sporadically (*n* = 15)	93.3		93.3		66.7		13.33		13.3		66.7		40	

* *p* ≤ 0.05, a: Chi-square, b: Fisher’s exact test; NA: Not applicable as there are no two categories of answers.

**Table 4:** Final model of multiple regression analysis of variables statistically associated with positivity for gastrointestinal parasites detected in fecal samples from pigs raised on family farms.

Information	Multivariate Logistic Regression	
	*p*-Value (*p* ≤ 0.05)	OR Adjusted (95% CI)
**Gastrointestinal parasite**		
Age range	0.0003	3.9153 (1.8810–8.1497)
**Form of the Phylum Ciliophora**		
Age range	0.0096	1.6960 (1.1372–2.5294)
**Coccidia oocysts**		
Food provided to pig	0.008	5.6971 (1.5761–20.593)
How to wash the environment	0.0391	5.4707 (1.0884–27.496)
Caring for the piglets	0.0004	3.9851 (1.8515–8.5773)
***Ascaris suum***		
Facilities type	0.0087	8.9037 (1.7374–45.6286)
**Strongyles**		
Age range	0.0109	1.7479 (1.1371–2.6870)
How to wash the environment	0.0469	8.6966 (1.0302–73.4132)
Floor of the buildings	0.0465	8.6587 (1.0341–72.4980)
***Strongyloides ransomi***		
Food provided to pig	0.0001	9.7365 (3.0710–30.8694)
Caring for the piglets	0.0004	3.6347 (1.7915–7.3744)
How to wash the environment	0.0253	11.0733 (1.3465–91.0613)

## Data Availability

Not applicable.

## Supplementary Materials

The following supporting information can be downloaded at: https://www.mdpi.com/article/10.3390/pathogens11090971/s1, Table S1: General information about pig-handling practices garnered from the questionnaire answered by 10 family-run pig farmers in Cachoeiras de Macacu, RJ; Table S2: General information about animal hygiene practices garnered from the questionnaire answered by pig farmers and their family members in Cachoeiras de Macacu, RJ.
