# Supplementary material for: Family-Run Pig Farms: Research and Extension Activities for Parasite Control in a Municipality in the State of Rio de Janeiro, Brazil
Source: Pathogens. 2022 Aug 26;11(9):971. doi: 10.3390/pathogens11090971 (PMC9503554; doi:10.3390/pathogens11090971)
Supplement: Supplementary file 1 [file pathogens-11-00971-s001.zip › pathogens-1799717-supplementary.pdf]

Table S1. General information about pig handling practices garnered from the questionnaire answered by 10 family-run pig farmers in Cachoeiras de Macacu, RJ.

Information	Responses in order of frequency					
	**1st place**	**%**	**2nd place**	**%**	**3rd place**	**%**
Property type	Little farm	7 (70%)	Backyard of the homes	3(30%)	-	-
Breed of the pig	Mixed breeds	9 (90%)	Mixed breeds and Duroc breed	1 (10%)	-	-
Type of creation	Confined in pig pen	8 (80%)	Confined in pig pen and free in the backyard	1 (10%)	Confined in pig pen and pig on a leash	1 (10%)
Stocking and categorization of animals by pig pen	There is no set number	5 (50%)	Divide pigs by age group	4 (40%)	Only one pig per pen	1 (10%)
Distance from the pigsty to the producer's house	10 to 100 meters	4 (40%)	More than 200 meters	2 (20%)	Residence in another location	2 (20%)
Facilities type	Collective bays with cement wall<br>Collective bays with cement wall, wooden or bamboo fence	4 (40%)<br>4 (40%)	Collective bays with cement and wood wall	2 (20%)	-	-
Pen roof	Totally covered with fiber cement tile, galvanized or PVC	9 (90%)	Partially covered with fiber cement tile	1 (10%)	-	-
Floor of the buildings	Naked soil or deteriorated cement	7 (70%)	Cemented or concreted	3 (30%)	-	-
Water to cool the pig	No	9 (90%)	Yes	1 (10%)	-	-
Habit of throwing water on the body of pigs to refresh them	Yes	10 (100%)	-	-	-	-
Supply of drinking water	*Ad libitum*	7 (70%)	Provided two or three times a day	3 (30%)	-	-
Type of drinking fountains	Cement fountain	4 (40%)	Nipple type	3 (30%)	Cement lame and plastic bowls	2 (20%)
Food provided to pig	Remains of human and agricultural food, wheat bran and/or barley	4 (40%)	Remains of human and agricultural food, maize or rice flour	3 (30%)	Agricultural remainder and specific pig feed and wheat bran<br>Horse feed, wheat bran, corn flour or rice and corn<br>Corn bran for pig	1 (10%)<br>1 (10%)
Frequency of feeding the pig	Twice a day	8 (80%)	Once a day	1 (10%)	Three times a day	1 (10%)
Feeder type	Cement feeder	6 (60%)	Floor and cement feeder	2 (20%)	Directly on the floor<br>Directly on the floor, plastic feeder and cut tire	1 (10%)
Food storage	Yes	8 (80%)	No	2 (20%)	-	-
Food storage location	Own room with ceiling	4 (40%)	Plastic drum	2 (20%)	Not store	2 (20%)
Type of food stored	Feed	4 (40%)	Food scraps	2 (20%)	Not store	2 (20%)
Other animals in the farms	Yes	10 (100%)	-	-	-	-
Sharing with other animal species	No	7 (70%)	Yes	3 (30%)	-	-
Observed rats on the property	Yes	8 (80%)	No	2 (20%)	-	-
Use of drug for rodent control	Yes	6 (60%)	No	4 (40%)	-	-
Presence of fly	Yes	6 (60%)	No	4 (40%)	-	-
Use of drug for fly control	No	6 (60%)	Yes	4 (40%)	-	-
Observed the pigs itch	Yes	5 (50%)	No	5 (50%)	-	-

Observed the pigs itch (team observation)	No	6 (60%)	Yes	4 (40%)	-	-
Noted blood on the pig feces	No	10 (100%)	-	-	-	-
Change in pig behavior	No	3 (30%)	Bite pigsty structure	3 (30%)	Cough	2 (20%)
Vaccinated animals	Yes	6 (60%)	No	4 (40%)	-	-
Anti - parasitc medicine	Yes	10 (100%)	-	-	-	-
Annti - ectoparasitic medicine	No	9 (90%)	Yes	1 (10%)	-	-
Reproduction carried out on the property	Yes	8 (80%)	No	2 (20%)	-	-
Type of reproduction performed	Hand mating	8 (80%)	Not reproduce	2 (20%)	-	-
Caring for the piglets	Breastfeeding after birth and/or teeth cutting	6 (60%)	Breastfeeding after birth, vaccination, iron supplementation and sterelization	4 (40%)	-	-
Accumulation of excreta in the pig enclosure (team observation)	No	6 (60%)	Yes	4 (40%)	-	-
Cleaning the enclosures	Manually with water	6 (60%)	Remove feces dry and use water	2 (20%)	Manually dry	1 (10%)
How to wash the environment	Water	9 (90%)	Not clean the environment, uses straw bedding	1 (10%)	-	-
Sanitary break	No	10 (100%)	-	-	-	-
Use of the flamethrower like to fire broom	No	10 (100%)	-	-	-	-
Cleaning utensils intended only for cleaning the pig facility	Yes	9 (90%)	No	1 (10%)	-	-
Specific clothing only for handling pigs	No	8 (80%)	Yes	1 (10%)	Sporadically	1 (10%)
	Closed boots and/or shoes and long pants	5 (50%)	Boots	2 (20%)	Not use	2 (20%)

- : there was no other response category.

Table S2. General information about animal hygiene practices garnered from the questionnaire answered by pig farmers and their family members in Cachoeiras de Macacu, RJ.

**Information**	Responses in order of frequency					
	1st place	%	2nd place	%	3rd place	%
Performed stool examination	Yes	26 (76.5%)	No	5 (14.7%)	Not remember	2 (5.9%)
Frequency of performing the stool test	Once a year	10 (29.4%)	Does not test for feces	8 (23.5%)	It's been a long time	6 (17.6%)
Anti-parasite medicine	Yes	28 (82.3%)	No	6 (17.6%)	-	-
Frequency of taking worm medicine	Once a year	9 (26.5%)	Don't take it often	7 (20.6%)	Never took medicine	4 (11.7%)
Intestinal pain	No	17 (50%)	Yes	16 (47%)	Seldom	1 (2.9%)
Frequency of belly pain	Seldom	17 (50%)	Sometimes	7 (20.6%)	Did not know how to answer	4 (11.7%)
Last report of diarrhea and intestinal upset	It's been a long time	12 (35.3%)	Not remember	10 (29.4%)	Did not know how to answer	6 (17.6%)
Frequency of diarrhea/intestinal disorder	Did not know how to answer	25 (73.5%)	Not remember	4 (11.7%)	Not often	3 (8.8%)
Pieces of helminth or whole helminth in their fezes	No	25 (73.5%)	Yes	9 (26.5%)	-	-
Observed blood in his stool	No	33 (97.1%)	Yes	1 (2.9%)	-	-
Residence water supply	Public piped water	18 (52.9%)	Spring water	13 (38.2%)	Artesian well	3 (8.8%)
Water treatment	Untreated	29 (85.3%)	Filtered	4 (11.7%)	Buy water	1 (2.9%)
Hygiene of roots and leafy vegetables	Washing with water	22 (64.7%)	Don't eat vegetables	3 (8.8%)	Washing waterm. vinegar and lemon	2 (5.9%)
Plantations at home	Yes	19 (55.9%)	No	15 (44.1%)	-	-
Plantations types	Fruits. vegetables and greens	15 (44.1%)	There is no plantation	15 (44.1%)	Only fruits	4 (11.7%)
Purpose of this plantation	Consumption	19 (55.9%)	There is no plantation	15 (44.1%)	-	-
Purpose of pig breeding	Consumption	16 (47%)	Consumption. exchange and sale	7 (20.6%)	Consumption and sale	6 (17.6%)
Frequency of pork consumption	Don't eat often	7 (20.6%)	Once a month	6 (17.6%)	Sometimes	5 (14.7%)
Pork point	Well done	29 (85.3%)	Don't eat pork	4 (11.7%)	Poorly done	1 (2.9%)
